# An improved rapid sampling microdialysis system for human and porcine organ monitoring in a hospital setting

**DOI:** 10.1039/c8ay01807c

**Published:** 2018-11-05

**Authors:** Sally A. N. Gowers, Karim Hamaoui, Natalie Vallant, George B. Hanna, Ara Darzi, Daniel Casanova, Vassilios Papalois, Martyn G. Boutelle

**Affiliations:** a Department of Bioengineering , Imperial College London , UK . Email: m.boutelle@imperial.ac.uk; b Department of Surgery & Cancer , Imperial College London , UK; c Department of Surgery , University of Cantabria , Santander , Spain

## Abstract

Online organ monitoring could provide clinicians with critical information regarding organ health prior to transplantation and could aid clinical decision-making. This paper presents the methodology of online microdialysis for real-time monitoring of human organs *ex vivo*. We describe how rapid sampling microdialysis can be incorporated with organ perfusion machines to create a robust organ monitoring system and demonstrate its use in monitoring human and porcine kidneys as well as human and porcine pancreases. In this paper we also show the potential usefulness of this methodology for evaluating novel interventions in a research setting. The analysis system can be configured either to analyse two analytes in one organ, allowing for ratiometric analysis, or alternatively to monitor one analyte in two organs simultaneously, allowing direct comparison. It was found to be reliable over long monitoring periods in real clinical use. The results clearly show that the analysis system is sensitive to differences between organs and therefore has huge potential as an *ex vivo* organ monitoring tool.

## Abstract 2

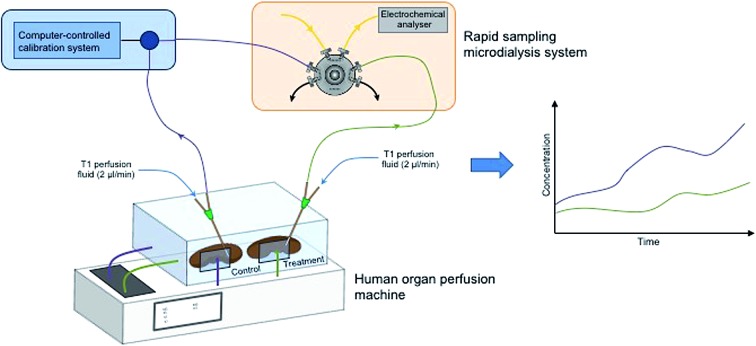
Online organ monitoring can provide clinicians with critical information regarding organ health prior to transplantation and could aid clinical decision-making.

## Introduction

Organ transplants can significantly improve or even save the lives of patients in need. For example, kidney transplantation is the treatment of choice for end-stage kidney disease as it considerably improves patient quality of life and reduces the need for long-term dialysis.^1^ However, there is a severe shortage of donor organs available for transplantation.^2^ As of March 2017, there were 4195 adult patients on the UK active kidney transplant list, compared to only 3042 adult kidney-only transplants carried out in the UK in 2016/2017.^3^ Current clinical practice is limited by the shortage of donor organs, their quality and the inability to assess their viability prior to transplantation.^4^,^5^ This shortage of available donor organs applies not only to kidneys but to other organs as well.

In order to expand the pool of donor organs, there has been increasing interest in utilising marginal donor organs, such donation-after-circulatory-death (DCD), which have historically not been used due to the risk of primary non-function and higher likelihood of delayed graft function as a result of longer warm ischaemia time (the time when the organ is warm and the blood supply has ceased).^1^,^6^ An alternative strategy to increase the number of available organs is to reduce the discard rate of expanded criteria donor (ECD) organs, such as those from donors who are aged 60 years or older or who are aged between 50 and 59 years and meet 2 of the following criteria: history of hypertension, serum creatinine level greater than 1.5 mg dl^–1^ (132.6 μM) and cerebrovascular cause of death.^7^ Despite increased risk of graft failure after an ECD kidney transplant,^7^ these transplants have been shown to be beneficial compared with remaining on dialysis.^8^

As a result of the move to use more DCD and ECD organs, there is an increasing need for better viability testing prior to transplantation in order to identify organs that are at risk of a poor outcome. Organ assessment may aid clinicians in deciding whether to accept or to discard a donor organ prior to transplantation, as well as allowing for possible intervention to improve the outcome. The period of time between retrieval and transplantation, in which organs are preserved, provides a unique opportunity to assess the health of the tissue.

Microdialysis is a tissue sampling technique that can be used to collect key markers of tissue health from the tissue of interest using a small sterile probe. The probe has a semi-permeable membrane at the tip and is perfused at low flow rates (0.1–2.0 μl min^–1^) creating a concentration gradient across the membrane. This results in the exchange of molecules between the tissue extracellular fluid and the probe, creating a dialysate stream that can be analysed for analytes of interest. Online microdialysis can be coupled to high-resolution analysis systems for *in vivo* monitoring.^9^–^13^ Microdialysis has been used with continuous biosensors or rapid online sampling in a wide range of applications to detect important information about tissue health and ischaemia.^14^–^21^ In situations where online analysis is not possible dialysate can be collected in lengths of low-volume tubing for analysis at a later time with retained temporal resolution.^22^

In order to implement microdialysis as part of an organ monitoring system the analysis system needs to be robust and be able to be integrated within the perfusion setup already established in the labs at Chelsea & Westminster Hospital. In addition, the system should be capable of being set up at short notice without preparation and should be amenable to being set up by non-experts. The opportunity to monitor human organs is incredibly rare and therefore valuable, so it is vital that the system is reliable and simple to use once installed. To meet these demands we set up an organ monitoring system consisting of two organ perfusion machines (Waters Medical) and a rapid sampling microdialysis (rsMD) online analysis system.

Previously, we have shown that rapid sampling microdialysis (rsMD) can be used to monitor *ex vivo* porcine kidneys during preservation.^17^ This demonstrated that microdialysis is a viable translational tool for donor organ monitoring and that the analysis technique is sensitive to metabolic differences between preserved kidneys. Changes seen were over a long time-scale, therefore, this analysis system is ideal for this application. rsMD can provide high-quality, well-controlled data and as a result, this method has great potential for use in monitoring human transplant organs. In addition, the system can be set up to monitor two organs simultaneously, making it ideal for human organ monitoring where multiple organs may be available at the same time, in order to maximise on this valuable data. In a clinical setting this method could be used to assess tissue health; in a research setting it could be useful in evaluating the effectiveness of various clinical interventions.

This paper presents the methodology for monitoring human and porcine kidneys during preservation and extends the technology to human and porcine pancreas monitoring. We present preliminary results of various interventions and demonstrate the potential usefulness of this tool to evaluate these measures.

## Experimental

### Organ retrieval

Human organs were supplied by NHS Blood & Transplant. Ethical approval for the use of human organs not suitable for transplantation was obtained through our study protocol 12/SW/202, IRAS project ID 8402 and was approved by the NRES Committee of South-West Exeter. After ethical approval was gained further approval was granted for use of the unused human grafts from NHS Blood & Transplant. All procedures were carried out in accordance with the Declaration of Helsinki.

Porcine organs were retrieved from a designated approved abattoir in accordance with our ethics approvals. The organs were retrieved by a clinician immediately after death to mimic a real clinical situation. In all cases the organs were immediately flushed with cold preservation solution, either University of Wisconsin (Viaspan, Bristol-Myers Squibb Pharmaceuticals Ltd, Ireland) solution (UW) or Soltran, until the effluent was clear. After this initial flush, the organs were stored in the flushing solution and transported on ice to the laboratory. Discarded human organs, which had been donated to scientific research were prepared according to normal clinical practice and brought to the laboratory on ice once they were made available to the clinical team.

### Organ microdialysis

**Fig. 1:**
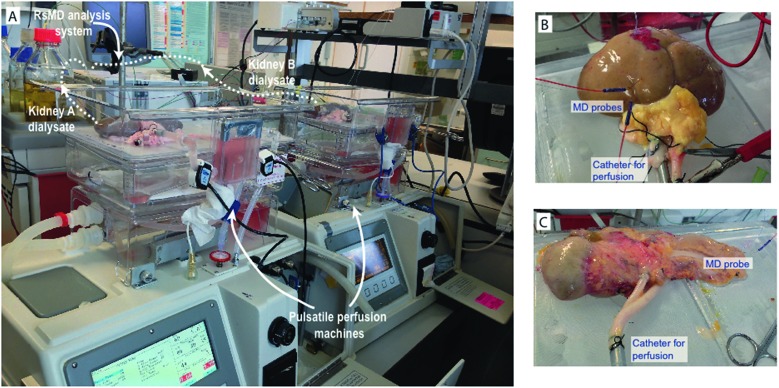
Setup for simultaneous organ perfusion and online monitoring at Chelsea & Westminster Hospital. (A) The complete setup consists of two perfusion machines (Waters Medical) for simultaneous perfusion of two organs and the online analysis system behind. The outlet of two microdialysis probes positioned in the organs can be fed into the analysis system for simultaneous measurement of both streams. (B) Perfusion of a discarded human kidney and (C) a discarded human pancreas on clinical perfusion machines. In both cases microdialysis (MD) probes were inserted in the tissue and connected to the rsMD analysis system for measurement of glucose and lactate in real time.

The organs were first connected to the perfusion machines, as shown in Fig. 1, prior to probe insertion. MAB 11.35.4 microdialysis probes (Microbiotech) with a 4 mm membrane and a 6 kDa molecular weight cut-off were used for these studies. For kidneys, the outer capsule was removed to help with probe insertion. A 21 G tunnelling needle was used to first make a hole in the tissue before microdialysis probe insertion. An alternative approach using a cannulation needle as a guide for probe insertion was tested, where the probe was threaded through the needle in the opposite direction to that in which the needle was inserted into the tissue. The needle was then removed leaving the probe in place, however, this procedure involved a large amount of manipulation of the kidney. In addition, in some cases multiple probes were inserted and this approach was only found to be suitable for the first probe insertion due to the amount of handling required. Fig. 1B and C show a human kidney and pancreas, respectively, connected to the perfusion machine, demonstrating how the microdialysis probes were positioned.

In all studies the microdialysis probe was perfused at 2 μl min^–1^ with T1 perfusion solution (2.3 mM calcium chloride, 147 mM sodium chloride, 4 mM potassium chloride) using a syringe pump. Probe perfusion commenced prior to insertion in order to verify that the probe was functioning correctly and none of the tubing was blocked before proceeding. As the organs were stationary throughout the experiments it was not necessary to suture the probes in place. All that was required to prevent the probes from moving was for the probe tubing to be taped to the side of the perfusion cassette.

### Online analysis system

The experimental setup for organ perfusion and monitoring is shown in Fig. 1A. The analysis part of the system needed to be integrated with the perfusion machines to form a complete system. Due to the unexpected nature of human organ availability it was important that the analysis system was robust and could be set up quickly without preparation. For kidneys, human organs were sometimes donated in pairs, therefore a system that was capable of monitoring both organs simultaneously was important in order to maximise the amount of data gathered from these rare opportunities. The system that was integrated into our organ perfusion and monitoring system was rapid sampling microdialysis (rsMD).

rsMD is a flow injection analysis system that combines sample collection and analysis and allows for sampling of metabolites with high temporal resolution in real time. This system has been used clinically to monitor changes in glucose and lactate levels in the brain^16^ and bowel^14^ during surgery and postoperatively^23^ and during free flap surgery.^18^ The dialysate stream is connected to a custom-made 6-port flow injection valve (Valco, Switzerland), which injects a sample of the dialysate into a stream of mediator and through the assay at regular intervals. We have moved away from the valve used in previously published papers as it was found to eventually fail after repeated use. For the work presented here we implemented a new 100 nl dual-internal loop Cheminert valve, which was found to be considerably more reliable than the previous design and therefore more suitable for this application.

**Fig. 2:**
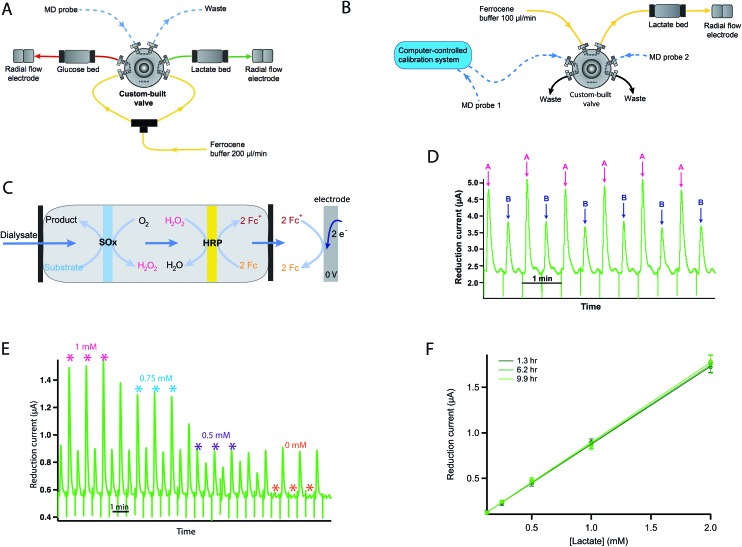
(A) rsMD setup for measurement of glucose and lactate in one sample stream. The microdialysis (MD) probe outlet is connected to the sample loop of the flow injection valve. Every 30 s a dialysate sample is injected alternately into the analysis flow stream for either glucose or lactate. (B) rsMD setup for measurement of lactate in two samples streams with autocalibration. The outlet of MD probe 1 is connected directly to the autocalibration board, which is connected to a loop of the flow injection valve, the outlet of MD probe 2 is connected to the opposite side of the analysis loop. Every 30 s a dialysate sample is injected into the analysis flow stream, alternating between each sample stream. (C) Reaction sequence occurring within the immoblised enzyme bed. The dialysate sample is mixed with the Fc mediator solution and is pumped through two membranes loaded with enzyme, the first with the SOx and the second with HRP. The enzymatic reactions result in the production of 2 ferrocenium ions (Fc^+^), which are detected at the glassy carbon electrode by reduction at 0 V. (D) Example of raw data peaks alternating between measurements from two different *ex vivo* organs, A and B. (E) Example of raw data for a 4-point lactate autocalibration, peaks alternating between the calibration stream (marked with an asterisk) and the second dialysate stream. (F) Example of repeated lactate calibration curves over a 10 hour monitoring period (at 1.3 h, 6.2 h and 9.9 h after monitoring began) showing long-term stability of the analysis method. Points are fitted with a straight line. Markers indicate the mean ± standard deviation for each measurement (*n* = 3).

The system is described in Fig. 2. It can be set up in two configurations depending on the application: the system can be configured to analyse one dialysate stream for two metabolites (Fig. 2A) or to analyse two dialysate streams for one metabolite only (Fig. 2B).

#### One sample, two analytes

In this configuration, the rsMD system can be used to monitor the dialysate from one microdialysis probe for levels of two analytes, in this case glucose and lactate. The outlet of the microdialysis probe is connected to the sample loop of the 6-port internal loop valve. A high-performance liquid chromatography (HPLC) pump (Flux Instruments, Switzerland) is used to flow a filtered ferrocene (Fc) mediator solution (1.5 mM ferrocene monocarboxylic acid, 1 mM ethylenediaminetetraacetic acid, 100 mM sodium citrate, 150 mM sodium chloride) at 200 μl min^–1^ into a T-connector, which splits the stream equally into two analysis loops of the valve, as shown in Fig. 2A. Every 30 seconds a dialysate sample is automatically injected alternately into one of the two analysis flow streams. Each analysis stream accelerates the dialysate through an enzyme reactor, containing the appropriate enzymes, to a downstream electrode, which detects a reduction current. The mechanism for this reaction is described in Fig. 2C.

#### Two samples, one analyte

This configuration was developed for use in the transplant kidney studies.^17^ In this configuration, the rsMD system can be used to simultaneously measure the metabolite concentration of one analyte in two analysis streams. In all cases where this configuration was used the metabolite that was measured was lactate. The outlet tubing of each microdialysis probe is connected to a separate sample loop of the flow injection valve, either side of the analysis loop, as shown in Fig. 2B. The Fc mediator solution is pumped by an HPLC pump at 100 μl min^–1^ into the analysis loop of the valve. Every 30 seconds a dialysate sample is injected into the analysis flow stream, alternating between the two dialysate streams. The dialysate sample is accelerated by the analysis stream through a lactate enzyme reactor to a downstream electrode that detects a reduction current. In this configuration it is possible to simultaneously measure analyte concentrations in dialysate streams from different tissues, for example from a pair of kidneys or from a porcine and human pancreas simultaneously, allowing direct comparison.

### Online assay

Dialysate samples are automatically injected into a Fc mediator flow stream and pumped through immobilised dual-enzyme reactors to a downstream glassy carbon working electrode for detection of glucose or lactate. These low-volume enzyme reactors consist of two nitrocellulose membranes (6 mm diameter discs), the first loaded with the substrate oxidase enzyme (SOx), either glucose oxidase (Sekisui, 1 mg ml^–1^ in Fc solution) or lactate oxidase (Sekisui, 2 mg ml^–1^ in Fc solution), and the second loaded with horseradish peroxidase (HRP, Sekisui, 0.5 mg ml^–1^ in Fc solution), held inside a reactor made from inline biocompatible filter components, comprising a stainless steel body and two polyether ether ketone (PEEK) filter end fittings (IDEX Health & Science, Germany).

The dialysate sample first passes through the SOx membrane, which catalyses the oxidation of the substrate, producing hydrogen peroxide. The hydrogen peroxide produced could be detected electrochemically but it would require high electrode potentials and would result in detection of other chemicals present in the dialysate.^24^ To overcome this issue, the second membrane loaded with HRP catalyses the reduction of hydrogen peroxide to water.^25^ The HRP is regenerated by oxidation of two Fc mediator species, producing ferrocenium ions, which are detected at the electrode by reduction, producing current peaks. The reaction sequence is shown in Fig. 2C. Fig. 2D shows exemplar data for lactate measurement in dialysate streams from two organs (A and B) simultaneously. Here the current peaks alternate between measurements for the two organs.

The electrodes used for detection of the substrate consist of a three-electrode system housed inside a radial flow cell (Unijet, BASi, USA). The flow from the enzyme reactor passes through the stainless steel jet counter electrode to the 3 mm diameter glassy carbon working electrode opposite. An Ag|AgCl reference electrode is embedded next to the working electrode. The working and counter electrodes are separated from each other by a 16 μm Teflon gasket. The flow injection valve, enzyme beds and potentiostats are positioned in a Faraday cage that can be positioned close to the transplant organ during preservation as shown in Fig. 1A.

### Assay calibration

The system was calibrated manually by injecting known concentrations of glucose/lactate standards into the flow injection valve. These injections result in current peaks, the amplitude of which is proportional to the concentration of substrate in the sample (due to the small loop volume). A calibration curve is constructed relating current to concentration for each substrate.

In order to automate this so that calibrations can be carried out without the need for someone to be present, an automatic calibration system was previously developed using LabSmith programmable components.^26^ This was used to improve the reliability of the results obtained as more regular calibrations were possible. This setup is shown in Fig. 2B. Here, the rsMD system was set up to measure the lactate concentration in two dialysate streams. The outlet of microdialysis probe 1 was connected to a valve on the autocalibration board and low-volume fluorinated ethylene propylene (FEP) tubing was used to connect the autocalibration board to the flow injection valve, while probe 2 was connected directly to another loop of the flow injection valve as normal. Fig. 2E shows an example of a calibration carried out using this system.

During calibrations, dialysate 1 was switched to a collection vial and the calibration stream was directed to the flow injection valve instead. As a result, peaks alternate between those for the calibration stream and those for dialysate 2. Once calibration was complete, dialysate 1 was directed to the flow injection valve. The autocalibration board has also been used in exactly the same way with the rsMD system in its other configuration, to analyse glucose and lactate (data not shown here).

Fig. 2F demonstrates the robustness of the system for monitoring over long periods of time as the calibration curve remains constant over 10 hours of monitoring.

### Data analysis

Data presented were collected using a PowerLab 8/30 data acquisition unit and LabChart software (ADInstruments, New South Wales, Australia) running on a Macbook portable computer (Apple, CA). Typically, reduction current peaks were inverted for convenience. Raw rsMD data were processed in MATLAB (MathWorks, USA) using algorithms previously developed in the group to remove common artefacts such as baseline ripples and spikes and to identify the peaks.^27^ When necessary, current peaks were separated at this stage to produce individual results for each organ. The current peaks were then converted into analyte concentrations using the appropriate calibration curve. Concentrations reported are dialysate concentrations and are not corrected for the unknown *in vivo* probe recovery.

### Offline measurements

In some experiments, where additional sample streams were required, dialysate was collected in fine-bore Portex tubing (0.4 mm internal diameter, Smiths Medical, UK) for offline analysis. We have previously shown that this is an effective methodology for high-value experiments and can be optimised for time resolution.^22^ This enabled samples to be collected for analysis at a later time while retaining temporal resolution. For flow rates of 2 μl min^–1^, 63 minutes of dialysate could be collected per 1 m length. The ends of each of these lengths of storage tubing were melted to seal them so that the dialysate samples were not lost. They were then stored in the freezer until they could be analysed. The original direction of flow was noted so that the tubing could be ‘played back’ as if in real time. Experiments to validate this technique are shown elsewhere in detail.^22^ Clearly temporal resolution is reduced by dispersion, however, this can be minimised by optimisation of the tubing size and length.^22^ In this case a *T*_90_ of 10 min was observed for a pulse of increased concentration.^22^

### Organ monitoring

A number of exemplar experiments with both human and porcine organs will be presented to demonstrate the potential of online microdialysis for monitoring organs *ex vivo*. Each experimental protocol will be described in detail in the following sections.

#### Discarded human kidney during preservation and reperfusion

A kidney was retrieved from a female donor (65–70 years old). The organ was not suitable for transplantation as the donor had hydronephrosis, a condition in which the kidney becomes swollen because of a build-up of urine. The organ had 20 minutes of warm ischaemia, after which it was flushed with preservation solution and stored on ice before monitoring began. CIT (cold ischaemia time) prior to monitoring was approximately 16.5 hours. The kidney was perfused with cold UW solution for 5 hours, followed by 2 hours of warm perfusion with oxygenated Krebs–Henseleit buffer to mimic reperfusion. A microdialysis probe was inserted superficially in the cortex prior to perfusion (shown in Fig. 1B) and perfused at 2 μl min^–1^ with T1 solution. The kidney was monitored using the online rsMD system, which formed part of the organ monitoring setup at Chelsea & Westminster Hospital, as shown in Fig. 1A. In this case the system was configured so that both glucose and lactate could be measured in the cortex in real time.

#### Effect of protein treatment – human kidneys

A pair of discarded kidneys was retrieved from a 59 year old male donor. One organ was not suitable for transplantation because of glomerulosclerosis and microthrombosis (B), while the other was suitable for transplantation but no suitable recipient could be found in time (A). Both organs were flushed with preservation solution and stored on ice for 78 hours prior to monitoring. This study aimed to investigate the effect of treatment with a novel cytotopic anticoagulant peptide, thrombalexin, which is thought to prevent thrombosis upon reperfusion.^28^ Kidney A was treated with the protein during cold perfusion, while kidney B was perfused with a control solution. After treatment with the protein, the kidneys were both perfused with porcine blood from the local abbatoir (as human blood was not available). A microdialysis probe was inserted superficially into the cortex of each kidney and perfused at 2 μl min^–1^ with T1 solution prior to perfusion. After the initial 4 hours of hypothermic machine perfusion (HMP), probes were removed and reinserted for the haemoperfusion phase. Cortical lactate dialysate levels in both kidneys were analysed online in real time using the rsMD analysis system.

#### Effect of protein treatment – porcine kidneys

To further investigate the effect of the protective anticoagulant peptide thrombalexin, the same protocol was repeated with two pairs of porcine kidneys. This has been described in detail elsewhere.^28^ Briefly, in each pair one kidney was treated with the novel protein and the other acted as its control. A microdialysis probe, perfused with T1 solution at 2 μl min^–1^, was inserted into the cortex of each kidney prior to perfusion. Dialysate was analysed online in real time for levels of lactate in the two kidneys using the rsMD analysis system.

#### Porcine pancreases

After organ retrieval and transport to the laboratory, pancreases underwent either 24 hours (i) or 48 hours (iii) of static cold storage (SCS). Following SCS, the pancreases were cold-perfused for an additional 5 hours on a Waters Medical Systems RM3 perfusion machine (30–50 mmHg) with in-house Belzer-UW solution, with added mannitol (60.4 mM) to reduce oedema, in an attempt to ‘recondition’ the tissue. After cold preservation, the pancreases were perfused with oxygenated blood at body temperature for 2 hours to mimic reperfusion. During haemoperfusion, any pancreatic juice that was produced was drained from the organ through a Foley catheter. After about 30 minutes of haemoperfusion, glucose was added into the blood so that the final blood glucose concentration was 22 mM. This was carried out to stimulate the pancreas and to test how well it was functioning. Blood measurements were also taken using a commercial hospital analyser to check the concentration of glucose in the blood. Probes were temporarily removed between HMP and haemoperfusion phases as the perfusion machines had to be prepared for the next stage. Dialysate from pancreas (iii) was analysed online using the rsMD system for levels of glucose and lactate. In the case of (i), the rsMD system was re-wired so that lactate could be measured in real time simultaneously for two organs.

#### Human pancreases

Discarded human pancreases were made available to the clinical team on two occasions. The same protocol was used as described for the porcine pancreases. Each pancreas was stored on ice before being subjected to 5 hours of HMP with in-house Belzer-UW solution, followed by 2 hours of reperfusion with warm oxygenated Krebs–Henseleit buffer (Sigma-Aldrich, UK). As with the porcine pancreases, the microdialysis probes were removed between the HMP and the reperfusion stages. Human pancreas (ii) was donated by a 62 year old man. The organ was offered for transplantation but was refused on the basis of donor age and the presence of atherosclerotic plaques. The pancreas was stored on ice for 25 hours before HMP commenced. In the case of human pancreas (iv), no information was available about the donor. The organ was offered for transplantation and was matched with a recipient, but the recipient was not fit for surgery and by that time the CIT was too long to match the organ to another recipient. The pancreas was stored on ice for 57 hours before HMP commenced. Fig. 1C shows a microdialysis probe positioned in a human pancreas during HMP.

## Results and discussion

rsMD has the important advantage that it can be easily configured to have a large dynamic range and is particularly stable over long periods of monitoring in real clinical use (Fig. 2F). In these studies the analysis system could be operated in one of two modes, allowing us either to measure two analytes in one organ or to monitor two organs for one analyte at the same time, depending on the experimental requirements. It was routinely possible to monitor organs for 24 hours continuously. The next sections will describe and discuss some of the measurements obtained from human and porcine organ *ex vivo* monitoring.

### Discarded human kidney during preservation and reperfusion

**Fig. 3:**
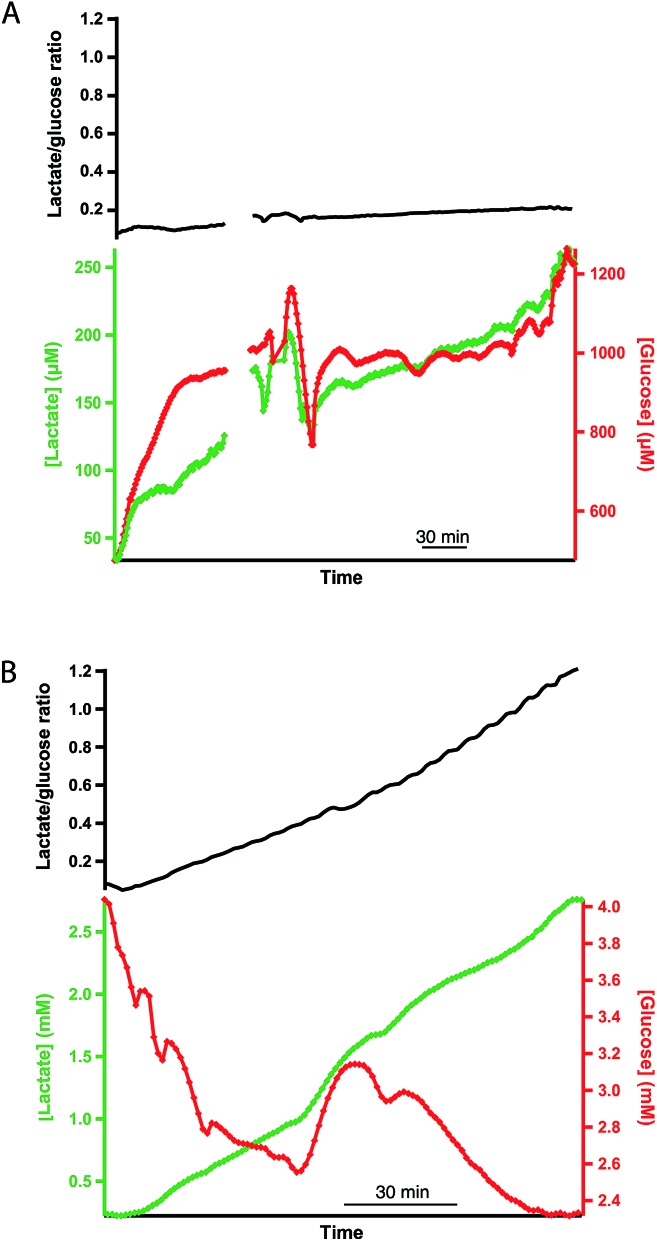
Dialysate cortical metabolite levels in a human kidney during (A) HMP and (B) warm reperfusion. The green trace shows dialysate lactate concentrations, the red trace shows dialysate glucose concentrations and the black trace shows the corresponding lactate/glucose ratio. Data were obtained in real time using rsMD, with a point every minute for each metabolite.

Dialysate glucose and lactate concentrations in the cortex of a human kidney are shown in Fig. 3A and B during hypothermic machine perfusion (HMP) and reperfusion, respectively. The steadily increasing lactate level observed is in close agreement with that seen in porcine kidneys during HMP.^17^ This result suggests that porcine kidneys provide a good model for human kidneys in research studies. While cortical lactate levels steadily increased during HMP, glucose levels initially increased and then seemed to stabilise after 1 hour of perfusion. The lactate/glucose ratio, which is typically a more sensitive marker for tissue health than either metabolic marker alone^18^ also increased throughout HMP.

During reperfusion, lactate levels were initially stable before increasing, whereas glucose levels decreased more steeply. The lactate/glucose ratio increased steadily throughout. This classic signature of ischaemia suggests that, despite the provision of nutrients and oxygen during reperfusion, anaerobic metabolism still dominated. There is a large peak in glucose levels midway through reperfusion that is completely removed in the lactate/glucose ratio at this point, suggesting that this is an artefact probably caused by a change in microdialysis probe recovery due to tissue changes. This demonstrates the value of making ratiometric measurements and that the system is tolerant even to changes in tissue property.

### Effect of protein treatment

**Fig. 4:**
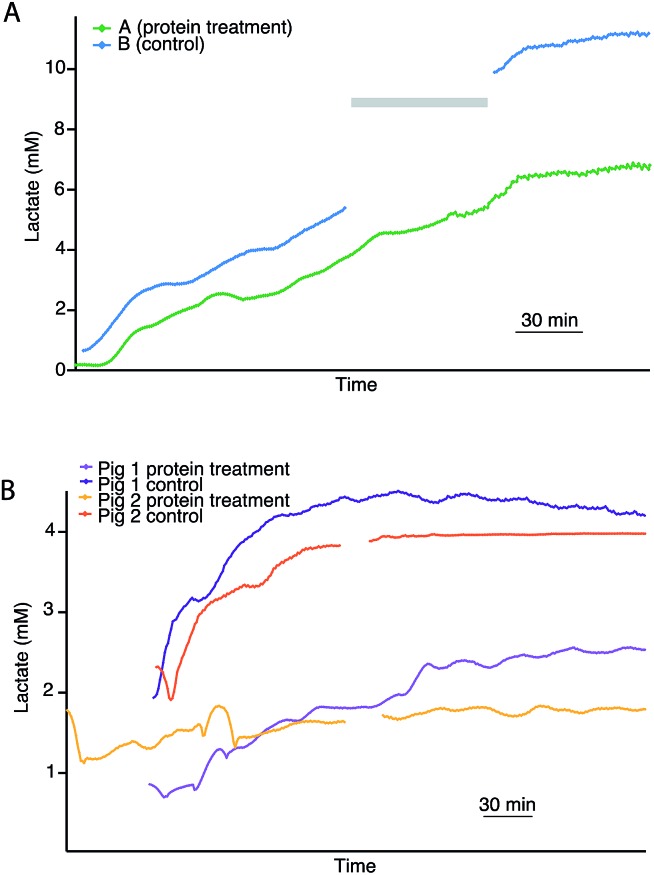
(A) Dialysate cortical lactate profiles during haemoperfusion in paired human kidneys with (A) and without (B) thrombalexin pre-treatment. Both kidneys were previously subjected to 4 hours of HMP, after which kidney A (green) was treated with the novel cytotopic anticoagulant peptide thrombalexin and kidney B (blue) acted as the control. Following protein treatment, both kidneys were perfused at body temperature. Due to technical issues lactate levels were not recorded for kidney B during the time period indicated by a grey bar. (B) Dialysate cortical lactate profiles of two pairs of porcine kidneys undergoing haemoperfusion with and without protein pre-treatment. The plateau in lactate after 3.5 h haemoperfusion in the orange trace is caused by assay gain saturation at higher lactate levels. Each line represents real-time data of an individual kidney with a point every minute, smoothed with a Savitsky–Golay 21-point filter.

As rsMD provides good quality, reliable data, it is possible to monitor pairs of kidneys in an attempt to investigate the potential effect of novel treatment interventions. Fig. 4A shows dialysate lactate levels in a pair of human kidneys during haemoperfusion. Kidney A was pre-treated with a novel cytotopic anticoagulant peptide and kidney B acted as its control. In both cases, cortical lactate levels steadily increased during haemoperfusion. The non-treated kidney showed considerably higher cortical lactate levels than its treated pair. However, in this case, the untreated kidney was not an ideal control as it was rejected from transplantation due to microthrombosis and therefore it is not possible to say whether these differences were due to the novel protein treatment or due to pre-existing differences between the two kidneys.

In order to investigate this further in a more tightly controlled comparison, the same protocol was carried out with pairs of porcine kidneys. Fig. 4B shows the dialysate lactate levels in two pairs of porcine kidneys treated in this way. In each pair, one kidney was treated with the novel proteins prior to haemoperfusion and the other acted as its control. For both pairs of kidneys, the kidney that had been pre-treated with the novel protein displayed considerably lower cortical lactate levels during than its non-treated pair during haemoperfusion. This is consistent with results obtained for human kidneys monitored using the same protocol (Fig. 4A). In all cases, the lactate levels initially increased before stabilising after about 3 hours.

During haemoperfusion there should be sufficient delivery of oxygen and glucose to the tissue to allow resumption of some degree of aerobic metabolism. Therefore, these preliminary experiments suggest that at the haemoperfusion stage the non-treated control kidneys were less healthy than their treated pairs, as the higher levels of cortical lactate observed suggest that anaerobic metabolism still dominated, possibly as a result of thrombosis upon reperfusion impairing the delivery of nutrients to the organ. Perfusion dynamics recorded using the Waters Medical perfusion machine support this interpretation as they showed higher perfusion flow indices and higher renal blood flow in the treated kidneys compared with the controls.^28^

### Human and porcine pancreases

**Fig. 5:**
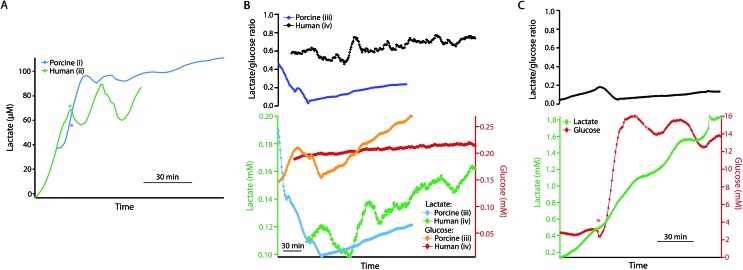
(A) Dialysate lactate levels in a porcine (i) and a human (ii) pancreas during haemoperfusion and reperfusion, respectively, following 24 or 25 h SCS and 5 h HMP. The porcine pancreas was perfused with blood and the human pancreas with oxygenated Krebs–Henseleit buffer at body temperature. The asterisk indicates the point at which additional glucose was added into the reperfusion solution, making the final concentration 22 mM. (B) Dialysate measurements for a porcine (iii) and a human (iv) pancreas during HMP following a long CIT (48 h and 57 h SCS). Dialysate glucose measurements are shown in yellow and red for the porcine and human pancreases, respectively, and dialysate lactate is shown in blue and green. The top trace shows the corresponding lactate/glucose ratio in for the porcine pancreas (iii) in blue and the human pancreas (iv) in black. (C) Dialysate glucose (red) and lactate (green) levels in human pancreas (iv) during reperfusion with warm oxygenated Krebs–Henseleit buffer following HMP. The red asterisk indicates the point at which additional glucose was added to the reperfusion solution, making the final concentration 22 mM. The top trace shows the corresponding lactate/glucose ratio. Data were obtained in real time using rsMD, with a point every minute.

The same rsMD setup was used to monitor both human and porcine pancreases *ex vivo* during preservation, as shown in Fig. 5. Upon haemoperfusion, all porcine and human pancreases monitored displayed signs of oedema and did not perfuse well. Moreover, the porcine organs did not produce bile or pancreatic juice nor was there any swelling of the small bowel; all of which indicate that the pancreases were not functioning as they should be, possibly as a result of the long CIT. The human organs in contrast produced a considerable amount of pancreatic juice and the duodenum visibly swelled because of this production, despite the oedema.

Fig. 5A shows the dialysate lactate levels in a porcine pancreas (i) during haemoperfusion following 24 h CIT and 5 h HMP, and in a human pancreas (ii) during haemoperfusion, following 25 h CIT and 5 h HMP. In both cases lactate levels initially increased. In each case, the asterisk indicates the point at which additional glucose was added into the reperfusion medium to make the final perfusate concentration 22 mM. In both cases dialysate lactate levels increased and then stabilized after addition of glucose to the perfusate. Interestingly, the lactate levels were considerably lower than normally observed in *ex vivo* kidneys throughout the reperfusion phase. Using the rsMD monitoring system allowed measurements to be made in porcine and human organs at the same time, allowing direct comparison between the two.

Fig. 5B shows the glucose and lactate levels for a porcine pancreas (iii) and a discarded human pancreas (iv) during 5 hours of HMP following a longer CIT (48 hours of SCS). In this case the porcine pancreas perfused very poorly with worsening oedema and swelling. Extra UW had to be added to the circuit after 1 hour of perfusion in order to maintain sufficient levels of perfusate. Finally, the experiment was terminated early because of the poor condition of the organ, therefore only HMP data is shown for this pancreas. Dialysate glucose and lactate levels are fairly similar between the human and porcine pancreases and in both cases both lactate and glucose levels increase throughout HMP.

Fig. 5C shows the dialysate glucose and lactate levels in human pancreas (iv) during reperfusion with oxygenated Krebs–Henseleit buffer, following the HMP phase. As with reperfusion of kidneys, dialysate lactate levels increased during pancreas reperfusion due to increased glycolysis as a result of the warm temperature and the supply of glucose. The lactate/glucose ratio was lower at this stage compared to during HMP due to higher levels of glucose during haemoperfusion. The asterisk indicates the point at which additional glucose was added into the reperfusion solution in order to stimulate the pancreas. This corresponded to a sharp increase in dialysate glucose levels and a continued increase in dialysate lactate levels. This change is also seen clearly as a decrease in the lactate/glucose ratio at this point. This clearly demonstrates that this monitoring technique allows us to resolve metabolic changes occurring in the tissue and to detect changes brought about by clinical interventions. These preliminary results suggest that rsMD could have potential for monitoring other transplant organs such as pancreases in addition to kidneys.

## Conclusions

In this paper we have demonstrated the potential of online microdialysis for human organ monitoring. We have shown that this methodology can provide useful information on the health of both human and porcine kidneys and pancreases, particularly by measuring the lactate/glucose ratio. In addition, we have demonstrated that the system is sensitive to differences between organs subjected to different treatments. Current research efforts in the group are focused on developing a portable online analysis system that is capable of monitoring organs throughout the journey from donation to transplantation.

## Conflicts of interest

There are no conflicts to declare.
